# Does self-sampling for human papilloma virus testing have the potential to increase cervical cancer screening? An updated meta-analysis of observational studies and randomized clinical trials

**DOI:** 10.3389/fpubh.2022.1003461

**Published:** 2022-12-08

**Authors:** Gianfranco Di Gennaro, Francesca Licata, Alessandro Trovato, Aida Bianco

**Affiliations:** Department of Health Sciences, School of Medicine, University of Catanzaro “Magna Græcia”, Catanzaro, Italy

**Keywords:** human papillomavirus, cervical cancer screening, self-sampling, uptake, acceptability, preference, systematic review, meta-analysis

## Abstract

**Objectives:**

A meta-analysis was conducted to examine the effectiveness of HPV self-sampling proposal on cervical cancer screening (CCS) uptake when compared with an invitation to have a clinician to collect the sample. Secondary outcomes were acceptability and preference of self-sampling compared to clinician-collected samples.

**Methods:**

The present systematic review and meta-analysis followed the Preferred Reporting Items for Systematic Reviews and Meta-Analyses (PRISMA) guidelines. Studies examining the CCS uptake comparing self-sampling over invitation to be sampled by an healthcare professional and examining the proportion of women accepting or preferring self-sampling vs. clinician-collected sampling were included. The CCS uptake was also explored according to strategy of self-samplers' distribution, collection device type and screening status. Peters' test and Funnel Plot inspection were used to assess the publication bias. Quality of the studies was assessed through Cochrane Risk of Bias and NIH Quality Assessment tools.

**Results:**

One hundred fifty-four studies were globally identified, and 482,271 women were involved. Self-sampling procedures nearly doubled the probability (RR: 1.8; 95% CI: 1.7–2.0) of CCS uptake when compared with clinician-collected samples. The opt-out (RR: 2.1; 95% CI: 1.9–2.4) and the door-to-door (RR: 1.8; 95% CI: 1.6–2.0) did not statistically significant differ (*p* = 1.177) in improving the CCS uptake. A higher relative uptake was shown for brushes (RR: 1.6; 95% CI: 1.5–1.7) and swabs (RR: 2.5; 95% CI: 1.9–3.1) over clinician-collected samples. A high between-studies variability in characteristics of sampled women was shown. In all meta-analyses the level of heterogeneity was consistently high (*I*^2^ > 95%). Publication bias was unlikely.

**Conclusions:**

Self-sampling has the potential to increase participation of under-screened women in the CCS, in addition to the standard invitation to have a clinician to collect the sample. For small communities door-to-door distribution could be preferred to distribute the self-sampler while; for large communities opt-out strategies should be preferred over opt-in. Since no significant difference in acceptability and preference of device type was demonstrated among women, and swabs and brushes exhibited a potential stronger effect in improving CCS, these devices could be adopted.

## Introduction

Genital infection with human papillomaviruses (HPV) is the most common sexually transmitted infection in the world (1). In some women, HPV infection will persist over time, and if this goes undetected and untreated, it can lead to precancerous cervical lesions and possibly progress to cervical cancer (2). HPV causes about 8.6% of the cancers affecting women worldwide. In absolute terms, about 570, 000 cases/year are estimated, almost all attributable to the HPV16/18 genotypes (3).

The time from HPV infection to cervical cancer will usually take 10–20 years or longer, and leaves great opportunity for screening and early detection (4). Indeed, secondary prevention measures such as cervical cytology (Pap smear), visual inspection with acetic acid or HPV testing, have strongly contributed to the reduction of incidence and mortality of cervical cancer, by identifying those women at high risk (5, 6). However, the adherence to screening programs in some areas of the world remains very low due to the invasiveness of the test and the lack of confidence in its effectiveness. Therefore, it is quite evident that the relevance of this public health issue necessitates innovative early detection approaches (7, 8). HPV testing through self-collected specimens has gained attention for its potential to increase screening participation. Recent systematic reviews have shown that high-risk HPV (hrHPV) testing on self-sampled specimens has a similar accuracy to detect underlying cervical precancer when compared to cytology on clinician-obtained cervical smears and under the condition that validated polymerase chain reaction (PCR)–based HPV assays are used (9, 10). In addition, several systematic reviews of randomized trials in the context of population-based screening programs showed that offering hrHPV self-sampling to never-screened and under-screened women increased participation compared with inviting women to have samples taken by healthcare professionals (HCPs) (11–13).

In recent years, numerous studies have investigated the acceptability of self-sampling methods (10, 14–16). Studies have considered women's attitudes toward self-collection and found that women have a high acceptance of and positive attitudes toward the use of self-collected HPV testing (9–11, 15, 16). Skepticism toward self-sampling has emerged, and it is attributable mainly to the fear of not carrying out a correct self-sampling or toward its underrated diagnostic performance (17, 18). Since the last published meta-analysis (19), several studies have measured the effectiveness of self-sampling in increasing the HPV-screening uptake. Moreover, it remains unclear which type of self-sampler offers a better performance. Therefore, we conducted an updated review and meta-analysis on women's attendance in cervical cancer screening (CCS) comparing self-sampled to clinician-collected specimens was conducted to assess whether the strategy of self-samplers' distribution (direct mailing to home, door-to-door distribution, or availability in clinics/pharmacies) and the type of device (brush, swab, lavage, tampon) and the screening status (never- or under-screneed vs. general population) could act as predictors of CCS uptake. Finally, the overall percentage of women who considered self-sampling to be acceptable and who preferred it over collection performed by healthcare personnel was estimated.

## Methods

The present systematic review and meta-analysis followed the Preferred Reporting Items for Systematic Reviews and Meta-Analyses (PRISMA) guidelines (20). The need for obtaining institutional review board approval or patient informed consent was waived for this study because it is a review of publicly available data.

### Protocol registration

This study was registered in the International Register of Systematic Reviews (PROSPERO 2021: CRD42021266637) and the protocol is available for download.

### Eligibility criteria

Studies were eligible if the following criteria were met: (1) examining the CCS uptake comparing self-sampling over invitation to be sampled by an HCP; (2) reporting enough data to estimate an effect size (Odds- or Risk-Ratio) of CCS uptake; (3) examining the proportion of women accepting or preferring self-sampling vs. clinician-collected sampling; (4) the study population involved women ages 18–70 years both among the general population and among those who were never- or under-screened; (5) the study was in English and published by May, 2022.

### Outcomes

The primary outcome was the CCS uptake comparing self-sampling with clinician-collected samples for HPV testing. The CCS uptake was also explored according to strategy of self-samplers' distribution, collection device type and screening status. Self-samplers' distribution strategies evaluated were door-to-door (i.e., self-samplers were directly distributed to women), opt-out (i.e., mailing self-sampling kits directly to women's home addresses) and opt-in (i.e., receiving an invitation to actively order the kit by phone, by ordinary mail, or by picking it up at the pharmacy or local clinics).

Secondary outcomes were acceptability and preference of self-sampling compared to clinician-collected samples. Acceptability was defined as a unique answer (yes/no) to questions like “Did you find self-sampling acceptable?”. Similarly to a previous meta-analysis, the proxy questions “Would you recommend self-sampling to a relative or friend of yours?” or “Would you be willing to use a self-sampler again in the future?” were taken into account (21). Studies in which acceptability was not reported as binary data but measured by a continuous or numerical ordinal variable (e.g., 0–10 scale) were not considered unless an acceptability cut off was established. With regard to the preference outcome, we considered studies in which, after using the self-sampler, women were asked whether they preferred self-sampling or clinician-collected samples for future HPV screening visits.

### Data sources and search strategy

A detailed bibliographic literature search was conducted until May 2022. Two co-authors (GDG, FL) independently searched PubMed, Web of Science, Scopus, Cochrane Central and Google Scholar combinations of the following keywords/Medical Subject Headings (MeSH) terms: “HPV”, “Human Papillomavirus”, “self-sampler”, “self-sampling”, “self-test”, “self-testing”, “home-based testing”, “community-based test”, “acceptability”, “acceptance”, “willingness”, “uptake”, “participation”, “preference”. Electronic searches were supplemented by manual searches of the reference list of relevant articles. Both observational and randomized studies were searched. Gray literature was not considered.

### Study selection

All articles retrieved from the systematic search were exported to the Mendeley reference manager (www.mendeley.com), wherein duplicates were sought and removed. Three authors (GDG, FL, AT) independently winnowed titles and abstracts of the candidate papers to make a first selection. Full-text of selected papers was read to assess their eligibility in terms of topics of interest and the target population. Disagreements were resolved through discussion with a third author (AB).

**Figure 1:**
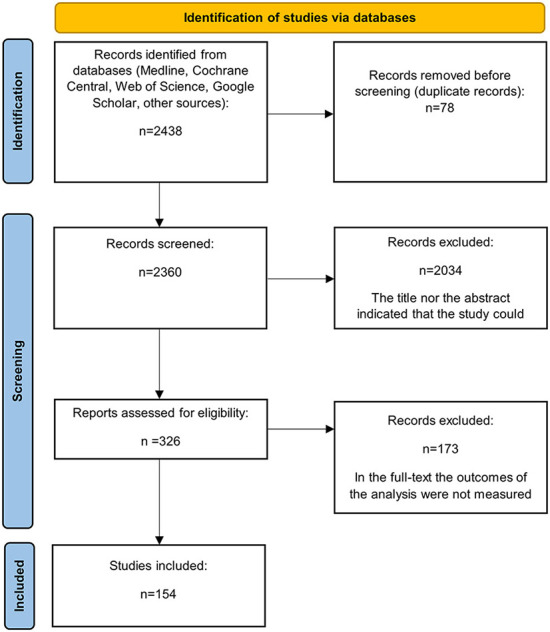
PRISMA flow chart of systematic review search process.

Relevant articles were reviewed in full if the study abstract met the inclusion criteria or if an article lacked sufficient information in the abstract to make an inclusion/exclusion judgement, to minimize errors of omission. Figure 1 summarizes the flow diagram of the literature search and the study selection process.

### Data extraction

An electronic collection form was used to extract the following information for each study: first author, year of publication, country, type of device (brush, swab, tampon or lavage), screening status (never or under-screened or general population), study design (observational or randomized). Women defined as “never-screened”, “under-screened”, “non-attendee” or “non-responders” to regular screening invitations were classified as “under-screened”. The self-samplers' distribution strategy (i.e., door-to-door, opt-out or opt-in strategy) was also retrieved. Regarding studies on acceptability and preference, information about the setting in which self-sampling occurred (at home or in a clinic) was also extracted.

### Quality assessment

Study quality was independently assessed by three authors (GDG, FL, AT) through the revised Cochrane Risk of Bias (RoB2). Tools for parallel and cluster-randomized trials or the National Institutes of Health (NIH). Quality Assessment Tool for Observational Cohort and Cross-Sectional Studies, depending on the study design (22, 23). The ratings (good, fair or poor methodological quality) assigned by each reviewer were compared and disagreements were discussed between the two reviewers. If consensus was not reached, a third reviewer (AB) arbitrated.

### Statistical analysis

As a primary analysis, the overall CCS uptake were pooled between distribution of self-samplers' and clinician-collected samples, using a DerSimonian and Laird random-effects model (24). Subgroup analyses were successively performed to assess whether differences in the CCS uptake were attributable to the self-samplers' distribution strategy, device type, women's screening status and study design (RCTs vs. observational). Relative Risks (RRs) were reported in the forest plots as measure of the effect size.

Secondary outcomes were analyzed by meta-analysis of proportions. Since outcome proportions were often higher than 80%, the confidence intervals were calculated through Freeman-Tukey double-arcsin transformation, and subsequently retro-transformed to avoid compression of standard errors and consequent biased results. The Wilson method was used to compute 95% Confidence Intervals (CIs). Subgroup analyses were performed to investigate whether brushes, swabs, tampons and lavages were equally accepted and whether the device category influenced the preference of self-sampling vs. outpatient sampling. A further subgroup analysis was performed to estimate the impact of the self-sampling setting (at home or in a clinic) on the acceptability or preference. Cochran's Q test was used to investigate overall differences between subgroups, while pair-wise comparisons (among self-samplers' distribution strategies and device types) were performed by contrasting meta-regression coefficients of models with one predictor only. *I*-squared consistency index was calculated to assess heterogeneity among studies. Peters' test and Funnel Plot inspection were used to assess the publication bias. To ensure the robustness of the results, subgroup analyses were repeated considering only RCTs. Data were analyzed by the statistical software STATA software, version 16.1 (25).

## Results

Databases searches yielded a total of 2, 438 articles, 78 of which were duplicates. Inspection of titles and abstracts resulted in the deletion of 2, 034 articles. A total of 326 full-text articles were retrieved for full review, and 154 articles met the inclusion criteria and were included in the analyses.

Overall, 482,271 women were involved, and all five continents were represented. Fifty-one (33.1%) studies were carried out in low-middle-income countries.

All but one of the RCTs showed a low risk of bias (Table 1). On the contrary, 53 (58.9%) out of 90 quasi-experimental or cross-sectional studies exhibited a fair or low overall quality (Table 2).

**Table 1:** Risk of bias of included RCTs assessed by Cochrane risk of bias tools.

**First authors**	**Year**	**Risk of bias arising from the randomization process**	**Risk of bias due to deviations from the intended interventions (effect of assignment to intervention)**	**Risk of bias due to deviations from the intended interventions (effect of adhering to intervention)**	**Risk of bias due to missing outcome data**	**Risk of bias in measurement of the outcome**	**Risk of bias in selection of the reported result**	**Overall risk of bias judgment**
Arrossi et al. (26)	2015	Some concerns	Some concerns	Low	Low	Low	Low	Low
Bais et al. (27)	2007	Low	Low	Low	Low	Low	Low	Low
Bosgraaf et al. (28)	2014	Low	Low	Low	Low	Low	Low	Low
Brewer et al. (29)	2021	Some concerns	Some concerns	Low	Low	Low	Low	Low
Broberg et al. (30)	2014	Some concerns	Low	Low	Low	Low	Low	Low
Cadman et al. (31)	2015	Low	Low	Low	Low	Low	Low	Low
Carrasquillo et al. (32)	2018	Low	Low	Low	Low	Low	Low	Low
Castle et al. (33)	2019	Some concerns	Some concerns	Low	Low	Low	Low	Low
Catarino et al. (34)	2015	Low	Low	Low	Low	Low	Low	Low
Darlin et al. (35)	2013	Some concerns	Low	Low	Low	Low	Some concerns	Low
Flores et al. (36)	2021	Low	Low	Low	Low	Low	Low	Low
Giorgi Rossi et al. (37)	2011	Low	Low	Low	Low	Low	Low	Low
Giorgi Rossi et al. (38)	2015	Low	Low	Low	Low	Low	Low	Low
Gizaw et al. (39)	2019	Low	Some concerns	Low	Low	Low	Low	Low
Gok et al. (40)	2010	Low	Low	Low	Low	Low	Low	Low
Gok et al. (41)	2012	Low	Low	Low	Low	Low	Some concerns	Low
Gustavsonn et al. (42)	2018	Low	Low	Low	Low	Low	Low	Low
Haguenor et al. (43)	2014	Low	Low	Low	Low	Low	Low	Low
Harper et al. (44)	2002	Low	Low	Low	Low	Low	Low	Low
Hellsten et al. (45)	2021	Low	Low	Low	Low	Low	Low	Low
Ivanus et al. (46)	2018	Low	Low	Low	Low	Low	Low	Low
Jalili et al. (47)	2019	Low	Low	Low	Low	Low	Low	Low
Karjalainen et al. (48)	2016	Low	Low	Low	Low	Low	Low	Low
Kellen et al. (49)	2018	high	Low	Low	Low	Low	Low	Low
Kitchener et al. (50)	2018	Low	Low	Low	Low	Low	Low	Low
Lazcano-Ponce et al. (51)	2011	Some concerns	Some concerns	Low	Low	Low	Some concerns	Some concerns
Lilliecreutz et al. (52)	2020	Low	Low	Low	Low	Low	Low	Low
Mac Donald et al. (53)	2021	Some concerns	Some concerns	Low	Low	Low	Low	Low
Modibbo et al. (54)	2017	Some concerns	Some concerns	Low	Low	Low	Some concerns	Low
Molokwu et al. (55)	2018	Low	Low	Low	Low	Low	Low	Low
Moses et al. (56)	2015	Low	Low	Low	Some concerns	Low	Low	Low
Murphy et al. (57)	2016	Low	Low	Low	Low	Low	Low	Low
Peeters et al. (58)	2020	Some concerns	Some concerns	Low	Low	Low	Low	Low
Polman et al. (59)	2019	Low	Low	Low	Low	Low	Low	Low
Racey et al. (16)	2016	Low	Low	Low	Some concerns	Low	Low	Low
Reques et al. (60)	2021	Some concerns	Low	Low	Some concerns	Low	Low	Low
Sancho-Garnier et al. (61)	2013	Some concerns	Some concerns	Low	Low	Low	Low	Low
Scarinci et al. (62)	2021	Low	Low	Low	Low	Low	Low	Low
Sewali et al. (63)	2015	Low	Low	Low	Low	Low	Low	Low
Sultana et al. (64)	2016	Low	Low	Low	Low	Low	Some concerns	Low
Szarewski et al. (65)	2011	Some concerns	Some concerns	Low	Low	Low	Low	Low
Tamalet et al. (66)	2013	Low	Low	Low	Low	Low	Low	Low
Tranberg et al. (67)	2018	Low	Low	Low	Low	Low	Low	Low
Van de Wijgert et al. (68)	2006	Low	Low	Low	Low	Low	Low	Low
Virtanen et al. (69)	2011	Some concerns	Low	Low	Low	Low	Low	Low
Virtanen et al. (70)	2015			Low				Low
Viviano et al. (71)	2017	Low	Low	Low	Low	Low	Low	Low
Wikstrom et al. (72)	2011	Some concerns	Some concerns	Low	Low	Low	Low	Low
Winer et al. (73)	2019	Low	Low	Low	Low	Low	Low	Low
Wong et al. (74)	2018	Low	Low	Low	Low	Low	Low	Low
Wong et al. (75)	2016	Low	Low	Low	Low	Low	Low	Low
Yamasaki et al. (76)	2019	Low	Low	Low	Low	Low	Low	Low
Zehbe et al. (77)	2016	Some concerns	Low	Low	Low	Low	Low	Low

**Table 2:** Risk of bias of included observational studies assessed by NIH Quality assessment tool for observational cohort and cross-sectional studies.

**First authors**	**Year**	**Research question clearly stated**	**Study population clearly specified and defined**	**Participation rate of eligible persons at least 50%**	**Eligibility criteria applied uniformly to all participants**	**Sample size justification, power description, or variance and effect estimates provided**	**Different level of exposure**	**Exposure clearly defined**	**Outcome measures clearly defined, valid, reliable and implemented consistently across all study participants**	**Key potential confounding variables measured and statistically adjusted**	**Overall quality**
Agorastos et al. (78)	2005	Yes	Yes	Yes	Yes	No	No	Yes	Yes	No	Fair
Aiko et al. (79)	2017	Yes	Yes	Yes	Yes	No	No	Yes	Yes	No	Fair
Allende et al. (80)	2019	Yes	Yes	Yes	Yes	No	No	Yes	Yes	No	Fair
Anderson et al. (81)	2017	Yes	Yes	Yes	Yes	No	No	Yes	Yes	Yes	Good
Anhang et al. (82)	2006	Yes	Yes	Yes	Yes	No	No	Yes	Yes	Yes	Good
Bansil et al. (83)	2014	Yes	Yes	No	Yes	No	No	Yes	Yes	No	Poor
Barbee et al. (84)	2010	Yes	Yes	Yes	Yes	No	No	Yes	Yes	No	Fair
Behnke et al. (85)	2020	Yes	Yes	Yes	Yes	No	No	Yes	Yes	No	Fair
Berner et al. (86)	2013	Yes	Yes	Yes	Yes	No	No	Yes	Yes	Yes	Good
Brewer et al. (87)	2019	Yes	Yes	No	Yes	No	Yes	Yes	Yes	No	Fair
Broquet et al. (88)	2015	Yes	Yes	Yes	Yes	No	No	Yes	Yes	No	Fair
Castell et al. (89)	2014	Yes	Yes	Yes	Yes	No	No	Yes	Yes	No	Fair
Catarino et al. (90)	2015	Yes	Yes	Yes	Yes	No	No	Yes	Yes	Yes	Good
Chatzistamatiou et al. (14)	2020	Yes	Yes	Yes	Yes	No	No	Yes	Yes	No	Fair
Chatzistamatiou et al. (91)	2017	Yes	Yes	Yes	Yes	No	No	Yes	Yes	No	Fair
Chou et al. (92)	2016	Yes	Yes	No	Yes	No	No	Yes	Yes	No	Poor
Crofts et al. (93)	2015	Yes	Yes	Yes	Yes	No	No	Yes	Yes	No	Fair
Crosby et al. (94)	2015	Yes	Yes	Yes	Yes	No	No	Yes	Yes	Yes	Good
Dannecker et al. (95)	2004	Yes	Yes	Yes	Yes	No	No	Yes	Yes	No	Fair
de Melo Kuil et al. (96)	2017	Yes	Yes	Yes	Yes	Yes	No	Yes	Yes	No	Good
Delerè et al. (97)	2011	Yes	Yes	Yes	Yes	No	No	Yes	Yes	No	Fair
Des marais et al. (98)	2019	Yes	Yes	Yes	Yes	No	Yes	Yes	Yes	No	Good
Desai et al. (99)	2020	Yes	Yes	Yes	Yes	No	No	Yes	Yes	No	Fair
Duke et al. (100)	2015	Yes	Yes	No	Yes	No	No	Yes	Yes	No	Poor
Dutton et al. (101)	2020	Yes	Yes	Yes	Yes	No	No	Yes	Yes	No	Fair
Dzuba et al. (102)	2002	Yes	Yes	Yes	Yes	No	No	Yes	Yes	Yes	Good
Esber et al. (103)	2018	Yes	Yes	Yes	Yes	No	No	Yes	Yes	No	Fair
Galbraith et al. (104)	2014	Yes	Yes	Yes	Yes	No	No	Yes	Yes	Yes	Good
Goldstein et al. (105)	2020	Yes	Yes	Yes	Yes	Yes	No	Yes	Yes	No	Good
Gottschlich et al. (106)	2019	Yes	Yes	Yes	Yes	No	No	Yes	Yes	Yes	Good
Gottschlich et al. (15)	2017	Yes	Yes	No	Yes	No	No	Yes	Yes	Yes	Fair
Guan et al. (107)	2012	Yes	Yes	Yes	Yes	No	No	Yes	Yes	Yes	Good
Haile et al. (108)	2019	Yes	Yes	Yes	Yes	No	No	Yes	Yes	No	Fair
Hinten et al. (109)	2017	Yes	Yes	Yes	Yes	No	No	Yes	Yes	No	Fair
Igidbashian et al. (110)	2011	Yes	Yes	Yes	Yes	No	Yes	Yes	Yes	No	Good
Ilangovan et al. (111)	2016	Yes	Yes	Yes	Yes	No	No	Yes	Yes	No	Fair
Islam et al. (112)	2020	Yes	Yes	Yes	Yes	No	No	Yes	Yes	Yes	Good
Jones et al. (113)	2012	Yes	Yes	Yes	Yes	No	No	Yes	Yes	No	Fair
Jones et al. (114)	2008	Yes	Yes	Yes	Yes	No	No	Yes	Yes	No	Fair
Katanga et al. (115)	2021	Yes	Yes	Yes	Yes	No	No	Yes	Yes	No	Fair
Ketalaars et al. (116)	2017	Yes	Yes	Yes	Yes	Yes	No	Yes	Yes	No	Good
Khanna et al. (117)	2007	Yes	Yes	Yes	Yes	No	No	Yes	Yes	Yes	Good
Khoo et al. (12)	2021	Yes	Yes	Yes	Yes	No	No	Yes	Yes	Yes	Good
Kilfoyle et al. (118)	2018	Yes	Yes	No	Yes	No	No	Yes	Yes	Yes	Fair
Kohler et al. (13)	2019	Yes	Yes	Yes	Yes	No	No	Yes	Yes	No	Fair
Landy et al. (119)	2022	Yes	Yes	Yes	Yes	No	No	Yes	Yes	Yes	Good
Laskow et al. (120)	2017	Yes	Yes	Yes	Yes	Yes	No	Yes	Yes	No	Good
Litton et al. (121)	2013	Yes	Yes	Yes	Yes	No	No	Yes	Yes	No	Fair
Lorenzi et al. (122)	2019	Yes	Yes	Yes	Yes	No	No	Yes	Yes	No	Fair
Ma'som et al. (123)	2016	Yes	Yes	Yes	Yes	No	No	Yes	Yes	Yes	Good
Madhivanan et al. (124)	2021	Yes	Yes	Yes	Yes	Yes	No	Yes	Yes	No	Good
Mahande et al. (125)	2021	Yes	Yes	Yes	Yes	Yes	No	Yes	Yes	No	Good
Malone et al. (126)	2020	Yes	Yes	No	Yes	No	No	Yes	Yes	No	Poor
Mandigo et al. (127)	2015	Yes	Yes	Yes	Yes	No	No	Yes	Yes	No	Fair
Mao et al. (128)	2017	Yes	Yes	Yes	Yes	No	No	Yes	Yes	No	Fair
Maza et al. (129)	2018	Yes	Yes	Yes	Yes	Yes	No	No	Yes	No	Fair
McLarty et al. (130)	2019	Yes	Yes	Yes	Yes	No	No	Yes	Yes	No	Fair
Mremi et al. (131)	2021	Yes	Yes	Yes	Yes	No	No	Yes	Yes	Yes	Good
Murchland et al. (11)	2019	Yes	Yes	Yes	Yes	Yes	No	Yes	Yes	Yes	Good
Nakalembe et al. (132)	2020	Yes	Yes	Yes	Yes	No	No	Yes	Yes	Yes	Good
Nelson et al. (133)	2015	Yes	Yes	Yes	Yes	No	No	Yes	Yes	No	Fair
Nobbenhuis et al. (134)	2002	Yes	Yes	Yes	Yes	No	No	Yes	Yes	No	Fair
Obiri-Yeboah et al. (135)	2017	Yes	Yes	Yes	Yes	Yes	No	Yes	Yes	No	Good
Oranratanaphan et al. (136)	2014	Yes	Yes	Yes	Yes	Yes	No	Yes	Yes	No	Good
Pantano et al. (137)	2021	Yes	Yes	Yes	Yes	No	No	Yes	Yes	No	Fair
Penaranda et al. (138)	2015	Yes	Yes	No	Yes	No	No	Yes	Yes	No	Poor
Reiter et al. (139)	2020	Yes	Yes	Yes	Yes	No	No	Yes	Yes	Yes	Good
Rosenbaum et al. (140)	2014	Yes	Yes	No	Yes	Yes	No	Yes	Yes	No	Fair
Sechi et al. (141)	2022	Yes	Yes	Yes	Yes	Yes	No	Yes	Yes	No	Good
Sellors et al. (142)	2000	Yes	Yes	Yes	Yes	No	No	Yes	Yes	No	Fair
Shin et al. (143)	2019	Yes	Yes	Yes	Yes	No	No	Yes	Yes	Yes	Good
Silva et al. (144)	2017	Yes	Yes	Yes	Yes	No	No	No	Yes	No	Poor
Surriabre et al. (145)	2017	Yes	Yes	Yes	Yes	No	No	No	Yes	No	Poor
Swanson et al. (146)	2018	Yes	Yes	Yes	Yes	Yes	No	Yes	Yes	Yes	Good
Szarewski et al. (147)	2007	Yes	Yes	Yes	Yes	No	No	Yes	Yes	Yes	Good
Taku et al. (148)	2020	Yes	Yes	Yes	Yes	No	No	Yes	Yes	No	Fair
Tan et al. (149)	2021	Yes	Yes	No	Yes	No	No	Yes	Yes	No	Poor
Tiiti et al. (150)	2021	Yes	Yes	Yes	Yes	No	Yes	Yes	Yes	Yes	Good
Torrado Garcia et al. (151)	2020	Yes	Yes	Yes	Yes	No	No	Yes	Yes	No	Fair
Torres et al. (152)	2018	Yes	Yes	Yes	Yes	No	No	Yes	Yes	No	Fair
Trope et al. (153)	2013	Yes	Yes	Yes	Yes	No	No	Yes	Yes	No	Fair
Van Baars et al. (154)	2012	Yes	Yes	Yes	Yes	No	No	Yes	Yes	No	Fair
Virtanen et al. (155)	2014	Yes	Yes	No	Yes	No	No	Yes	Yes	No	Poor
Waller et al. (17)	2006	Yes	Yes	Yes	Yes	No	No	Yes	Yes	No	Fair
Wang et al. (156)	2020	Yes	Yes	Yes	Yes	Yes	Yes	Yes	Yes	No	Good
Wedisinghe et al. (157)	2022	Yes	Yes	Yes	Yes	No	Yes	Yes	Yes	No	Good
Wikstrom et al. (158)	2007	Yes	Yes	Yes	Yes	No	No	Yes	Yes	No	Fair
Winer et al. (159)	2016	Yes	Yes	Yes	Yes	No	Yes	Yes	Yes	Yes	Good
Wong et al. (160)	2020	Yes	Yes	Yes	Yes	No	No	Yes	Yes	Yes	Good
Zehbe et al. (161)	2011	Yes	Yes	Yes	Yes	No	No	Yes	Yes	No	Fair

### Cervical cancer screening uptake

Forty-nine (31.8%) of studies included measured CCS uptake (Table 3); 46 (93.9%) were RCTs and 3 (5.1%) were quasi-experimental studies. Regarding characteristics of the studied population, 40 studies (81.6%) were focused on under-screened women, while 9 (18.4%) involved the general population. Cervical brushes were used in 21 (42.9%) studies, swabs in 20 (40.8%) studies and lavages in 7 (14.3%) studies. In 3 (6.1%) studies, the type of device was not reported. In 2 (4.1%) studies, both a brush and a lavage were proposed to the participants. In 12 (24.5%) studies self-samplers were directly distributed to women (door-to-door), and the opt-out and opt-in strategies were used in 30 (61.2%) and 10 (20.4%) studies, respectively. In 7 (14.3%) studies both opt-out and opt-in strategies were examined.

**Table 3:** Characteristics of the included studies assessing cervical cancer screening (CCS) uptake comparing self-sampling with clinician-collected samples for HPV testing.

**First authors**	**Year**	**Country**	**Sample size**	**Design**	**Area**	**Sample age**	**Country economic status**	**Social subgroup**	**Screening status**	**Device type**	**Control**	**Intervention**	**Control arm size**	**Experimental arm size**
Arrossi et al. (26)	2015	Argentina	7, 650	Cluster randomized clinical trial	Urban and rural	40–49^#^	MIC	–	Under-screened	Brush	Door-to-door recommendation to have a clinician-collected sample	Door-to-door distribution of self-samplers by HCPs	4, 018	3, 632
Bais et al. (27)	2007	Netherlands	2, 830	Randomized clinical trial	Urban	30–50^§^	HIC	–	Under-screened	Brush	Reminder letter proposing a clinician-collected sample	Self-samplers mailed to home	284	2, 546
Brewer et al. (29)	2021	New Zeland	3, 553	Randomized clinical trial	Urban and rural	44^#^	HIC	Indigenous Māori, Pacific and Asian women	Under-screened	Swab	Invitation letter proposing a clinician-collected sample	Intervention 1: invitation letter proposing a self-sample at local hospital Intervention 2: self-samplers mailed to home	512	Intervention 1: 1, 574 Intervention 2: 1, 467
Broberg et al. (30)	2014	Sweden	8, 800	Randomized clinical trial	Urban and rural	46.8**	HIC	–	Under-screened	Brush	Control 1: reminder letter proposing a clinician-collected sample Control 2: reminder letter and reminder phone call proposing a clinician-collected sample	Self-samplers mailed to home	Control 1: 4, 000 Control 2: 4, 000	800
Cadman et al. (31)	2015	England	6, 000	Randomized clinical trial	Urban and rural	40.0*	HIC	–	Under-screened	Swab	Reminder letter proposing a clinician-collected sample	Self-samplers mailed to home	3, 000	3, 000
Carrasquillo et al. (32)	2018	USA	601	Randomized clinical trial	Urban and rural	48.7*	HIC	Ethnic minorities in South-Florida. Haitian, hispanic and black women	Under-screened	Swab	Control 1: outreach programme by HCPs proposing a clinician-collected sample Control 2: facilitated navigation by HCPs to have a clinician-collected sample	Health education programme with door-to-door distribution of self-samplers or facilitated navigation to Pap smear offered by HCWs	Control 1: 182 Control 2: 212	207
Castle et al. (33)	2019	Brazil	483	Randomized clinical trial	Urban	42.5**	MIC	–	Under-screened	Brush	Door-to-door proposal to have a clinician-collected sample	Intervention 1: door-to-door choice between self-sampling and Pap-testing by HCWs Intervention 2: door-to-door distribution of self-samplers by HCWs	160	Intervention 1: 162 Intervention 2: 161
Castle et al. (162)	2011	USA	119	Quasi-experimental trial	Rural	42.5**	HIC	Underserved women in the Mississippi Delta	Under-screened	Brush	Voucher for free and facilitated clinician-collected sample	Health education programme and door-to-door distribution of self-samplers by HCWs	42	77
Darlin et al. (35)	2013	Sweden	1, 500	Randomized clinical trial	Urban and rural	50.3**	HIC	–	Under-screened	Swab	Invitation and recall letter proposing a clinician-collected sample	Self-samplers mailed to home	500	1, 000
Duke et al. (100)	2015	Canada	6, 057	Quasi-experimental trial	Rural	45–49^†^	HIC	–	General population	Swab	Control 1: Promotion campaign and invitation letter proposing a clinician-collected sample Control 2: invitation letter proposing a clinician-collected sample	HPV screening promotion campaign and self-samplers available at public locations (i.e., hair salons, pharmacies)	Control 1:2, 761 Control 2: 1, 536	1, 760
Elfström et al. (163)	2019	Sweden	8, 000	Randomized clinical trial	Urban and rural	47.0*	HIC	–	Under-screened	Swab	Invitation letter proposing a clinician-collected sample	Intervention 1: invitation to order a self-sampler through an online application Intervention 2: self-samplers mailed to home	2, 000	Intervention 1: 2, 000 Intervention 2: 2, 000 Intervention 3: 2, 000
Enerly et al. (164)	2016	Norway	3, 393	Randomized clinical trial	Urban	35–49^†^	HIC	–	Under-screened	Brush/Lavage	Reminder letter proposing a clinician-collected sample	Self-samplers mailed to home	2, 593	800
Giorgi Rossi et al. (37)	2011	Italy	2, 473	Randomized clinical trial	Urban and rural	25–64^§^	HIC	–	Under-screened	Lavage	Control 1: reminder letter proposing a clinician-collected sample (HPV test) Control 2: reminder letter proposing a clinician-collected sample (PAP test)	Intervention 1: invitation to order a self-sampler by phone-call Intervention 2: self-samplers mailed to home	Control 1: 616 Control 2: 619	Intervention 1: 622 Intervention 2: 616
Giorgi Rossi et al. (38)	2015	Italy	14, 041	Randomized clinical trial	Urban and rural	30–64^§^	HIC	–	Under-screened	Lavage	Recall letter proposing a clinician-collected sample	Intervention 1: self-samplers mailed to home Intervention 2: self-samplers available at local pharmacies	5, 012	Intervention 1: 4, 516 Intervention 2: 4, 513
Gizaw et al. (39)	2019	Ethiopia	2, 356	Cluster randomized clinical trial	Urban and rural	30–34^†^	LIC	–	Under-screened	Brush	Community education programme proposing a clinician-collected sample	Community health education programme and invitation to self-sample at local hospital	1, 143	1, 213
Gok et al. (41)	2012	Netherlands	26, 409	Randomized clinical trial	Urban and rural	39–43^†^	HIC	–	Under-screened	Brush	Reminder letter proposing a clinician-collected sample	Self-samplers mailed to home	264	26, 145
Gok et al. (40)	2010	Netherlands	28, 073	Randomized clinical trial	Urban and rural	30–60^§^	HIC	–	Under-screened	Lavage	Reminder letter proposing a clinician-collected sample	Self-samplers mailed to home with previous notification	281	27, 792
Gustavsonn et al. (42)	2018	Sweden	36, 390	Randomized clinical trial	Urban and rural	39.5**	HIC	–	Under-screened	Brush	Reminder letter proposing a clinician-collected sample	Self-samplers mailed to home	18, 393	17, 997
Haguenor et al. (43)	2014	France	5, 998	Randomized clinical trial	Urban and rural	51.1*	HIC	–	Under-screened	Swab	Control 1: invitation letter proposing a clinician-collected sample Control 2: reminder letter and phone call proposing a clinician-collected sample	Self-samplers mailed to home	Control 1:1, 999 Control 2: 2, 000	1, 999
Hellsten et al. (45)	2021	Sweden	29, 604	Randomized clinical trial	Urban and rural	37.8**	HIC	–	General population	Swab	Invitation letter proposing a clinician-collected sample	Self-samplers mailed to home	14, 839	14, 765
Ivanus et al. (46)	2018	Slovenia	26, 556	Randomized clinical trial	Urban and rural	49.8*	HIC	–	Under-screened	Not Reported	Reminder letter proposing a clinician-collected sample	Intervention 1: self-samplers mailed to home Intervention 2: self-samplers available at local pharmacies	2, 600	Intervention 1: 9, 556 Intervention 2: 14, 400
Jalili et al. (47)	2019	Canada	1, 052	Randomized clinical trial	Urban and rural	42.6**	HIC	–	Under-screened	Brush	Invitation letter proposing a clinician-collected sample	Self-samplers mailed to home	523	529
Kellen et al. (49)	2018	Belgium	35, 895	Randomized clinical trial	Urban and rural	50–54^†^	HIC	–	Under-screened	Brush	Control 1: reminder letter proposing a clinician-collected sample Control 2: reminder letter and phone call proposing a clinician-collected sample	Intervention 1: invitation to order a self-sampler by phone-call or email Intervention 2: self-samplers mailed to home	Control 1: 8, 849 Control 2: 8, 830	Intervention 1: 9, 098 Intervention 2: 9, 118
Kitchener et al. (50)	2018	UK	8, 849	Cluster randomized clinical trial	Urban and rural	Not available	HIC	–	Under-screened	Brush and lavage	Control 1: invitation letter proposing a clinician-collected sample Control 2: nurse navigators proposing a clinician-collected sample Control 3: timed-appointment to have a clinician-collected sample	Intervention 1: self-samplers mailed to home Intervention 2: self-samplers available on request	Control 1: 3, 782 Control 2: 1, 007 Control 3: 1, 629	Intervention 1: 1, 141 Intervention 2: 1, 290
Landy et al. (119)	2022	UK	784	Randomized clinical trial	Urban	55–59^†^	HIC	–	General population	Swab	Invitation letter proposing a clinician-collected sample	Invitation letter proposing a clinician-collected sample or a self-sampler mailed to home	391	393
Lazcano-Ponce et al. (51)	2011	Mexico	22, 102	Randomized clinical trial	Urban and rural	35–39^†^	MIC	–	General population	Brush	Door-to-door education programme proposing a clinician-collected sample	Health education programme and door-to-door distribution of self-samplers by HCWs	12, 731	9, 371
Lilliecreutz et al. (52)	2020	Sweden	9, 752	Randomized clinical trial	Urban and rural	30–64^§^	HIC	–	Under-screened	Swab	Control 1: phone call proposing a clinician-collected sample Control 2: invitation letter proposing a clinician-collected sample	Self-samplers mailed to home	Control 1: 3, 146 Control 2: 3, 538	3, 068
Mac Donald et al. (53)	2021	New Zealand	1, 539	Cluster randomized clinical trial	Urban and rural	40–49^†^	HIC	–	Under-screened	Swab	Texting, email, letter or phone call proposing a clinician-collected sample	Self-samplers offered during a clinical visit	806	733
Modibbo et al. (54)	2017	Nigeria	400	Randomized clinical trial	Urban and rural	40.8*	MIC	–	General population	Swab	Invitation letter proposing a clinician-collected sample	Self-samplers mailed to home	200	200
Moses et al. (56)	2015	Uganda	500	Randomized clinical trial	Urban	39.1*	LIC	–	General population	Swab	Door-to-door appointment with HCWs proposing a clinician-collected sample	Door-to-door distribution of self-samplers by HCWs	250	250
Murphy et al. (57)	2016	USA	94	Randomized clinical trial	Urban	48.7*	HIC	HIV-positive women	Under-screened	Brush	clinician-collected sample proposed during a clinical visit	Self-samplers offered during a clinical visit	31	63
Peeters et al. (58)	2020	Belgium	88	Randomized clinical trial	Urban and rural	45–54^†^	HIC	–	Under-screened	Brush	Face-to-face general practitioner advice for a clinician-collected sample	Self-samplers offered face-to-face by general practitioner	43	45
Polman et al. (59)	2019	Netherlands	16, 361	Randomized clinical trial	Urban and rural	45.6**	HIC	–	General population	Brush	Invitation letter proposing a clinician-collected sample	Self-samplers mailed to home	8, 168	8, 193
Racey et al. (16)	2016	Canada	818	Randomized clinical trial	Rural	51.2**	HIC	–	Under-screened	Swab	Control 1: no intervention (opportunistic screening of women previously invited to have a clinician-collected sample) Control 2: invitation letter proposing a clinician-collected sample	Self-samplers mailed to home	Control 1: 152 Control 2: 331	335
Reques et al. (60)	2021	France	687	Randomized clinical trial	Urban	41.0*	HIC	Underprivileged women (sex workers, slum dwellers)	Under-screened	Not Reported	clinician-collected sample proposed during a clinical visit in a community setting	Self-samplers offered during a medical consultation in a community setting	304	383
Sancho-Garnier et al. (61)	2013	France	18, 730	Randomized clinical trial	Urban	40–44^†^	HIC	Women belonging to lower socio-economic groups	Under-screened	Swab	Reminder letter proposing clinician-collected sample proposed during a clinical visit	Self-samplers mailed to home	9, 901	8, 829
Scarinci et al. (62)	2021	USA	335	Cluster randomized clinical trial	Rural	43.0*	HIC	–	Under-screened	Brush	Door-to door invitation to have a clinician-collected sample	Door-to-door choice between self-sampling and Pap-screening	170	165
Sewali et al. (63)	2015	USA	63	Randomized clinical trial	Urban	55.1*	HIC	Somali immigrant women in Minnesota	Under-screened	Brush	Door-to door invitation to have a clinician-collected sample	Door-to-door distribution of self-samplers	31	32
Sultana et al. (64)	2016	Australia	8, 160	Randomized clinical trial	Urban and rural	40–49^†^	HIC	–	Under-screened	Swab	Invitation letter proposing a clinician-collected sample	Self-samplers mailed to home	1, 020	7, 140
Szarewski et al. (65)	2011	England	3, 000	Randomized clinical trial	Urban	48.0*	HIC	–	Under-screened	Swab	Reminder letter proposing a clinician-collected sample	Self-samplers mailed to home	1, 500	1, 500
Tamalet et al. (66)	2013	France	8, 081	Randomized clinical trial	Urban	45–54^†^	HIC	–	General population	Swab	Reminder letter proposing a clinician-collected sample	Self-samplers mailed to home	4, 314	3, 767
Tranberg et al. (67)	2018	Denmark	9, 791	Randomized clinical trial	Urban and rural	40–49^†^	HIC	–	Under-screened	Brush	Reminder letter proposing a clinician-collected sample	Intervention 1: self-samplers mailed to home Intervention 2: invitation (email, phone, text message) to order a self-sampler	3, 262	Intervention 1: 3, 265 Intervention 2: 3, 264
Virtanen et al. (69)	2011	Finland	1, 0014	Randomized clinical trial	Urban	42.2**	HIC	–	Under-screened	Lavage	Reminder letter proposing a clinician-collected sample	Intervention 1: self-samplers mailed to home after further invitation to Pap screening Intervention 2: self-samplers mailed to home with no further invitation letter	6, 302	Intervention 1: 1, 315 Intervention 2: 2, 397
Virtanen et al. (70)	2015	Finland	7, 552	Quasi-experimental trial	Urban	45–49^†^	HIC	–	Under-screened	Lavage	Reminder letter proposing a clinician-collected sample	Self-samplers mailed to home	7, 397	155
Viviano et al. (71)	2017	Switzerland	667	Randomized clinical trial	Urban	42.2**	HIC	–	Under-screened	Swab	Invitation letter proposing a clinician-collected sample	Self-samplers mailed to home	331	336
Wikstrom et al. (72)	2011	Sweden	4, 060	Randomized clinical trial	Urban	39–60^§^	HIC	–	Under-screened	Brush	Invitation letter proposing a clinician-collected sample	Self-samplers mailed to home (2, 000)	2, 060	2, 000
Winer et al. (73)	2019	USA	19, 851	Randomized clinical trial	Urban	50–54^†^	HIC	–	Under-screened	Not Reported	Invitation letter proposing a clinician-collected sample	Self-samplers mailed to home	9, 891	9, 960
Yamasaki et al. (76)	2019	Japan	249	Randomized clinical trial	Rural	40–49^†^	HIC	Women living on the remote Goto island	Under-screened	Brush	Reminder letter proposing a clinician-collected sample	Self-samplers mailed to home	124	125
Zehbe et al. (77)	2016	Canada	1, 002	Cluster randomized clinical trial	Rural	25–69^§^	HIC	–	General population	Swab	Community educational programme proposing a clinician-collected sample	Self-samplers mailed to home	598	404

Sample age reported as

* mean,

** weighted mean,

# median,

^*##*^weighted median,

† median age group or

§ range.

Country economic status reported as: HIC, high income country; MIC, middle income Country; LIC, low income country.

**Figure 2:**
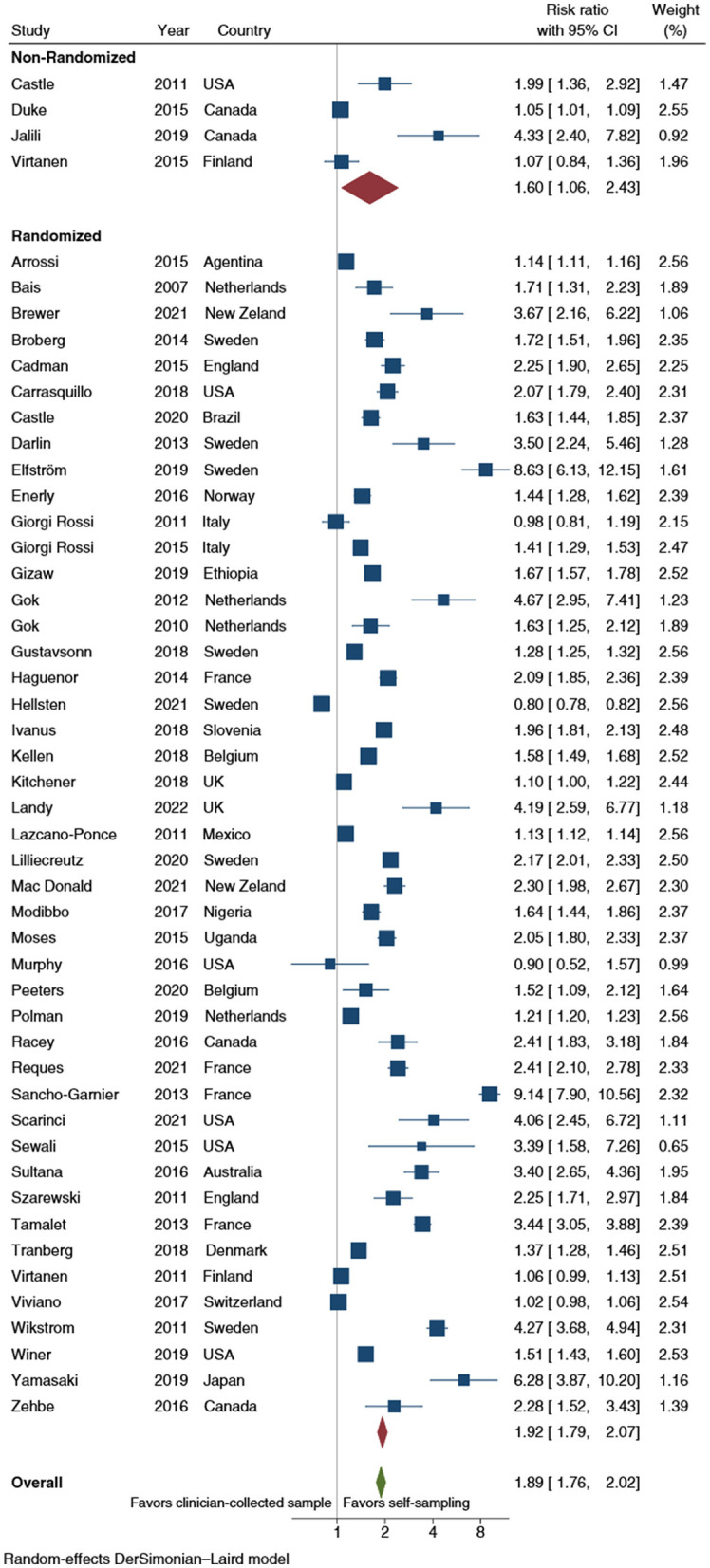
Forest plot comparing cervical cancer screening (CCS) uptake for HPV testing by self-sampling vs. clinician-collected samples, subgrouped by study design (randomized vs. non-randomized). Homogeneity: *I*^2^ = 98.9%; Cochrane's Q test for between-group differences: *Q* = 4,241.88; df = 1; *p* = 0.399.

Overall, self-sampling procedures nearly doubled the probability (RR: 1.9; 95% CI: 1.8–2.0) of CCS uptake when compared with clinician-collected samples (Figure 2).

#### Self-samplers' distribution strategy

**Figure 3:**
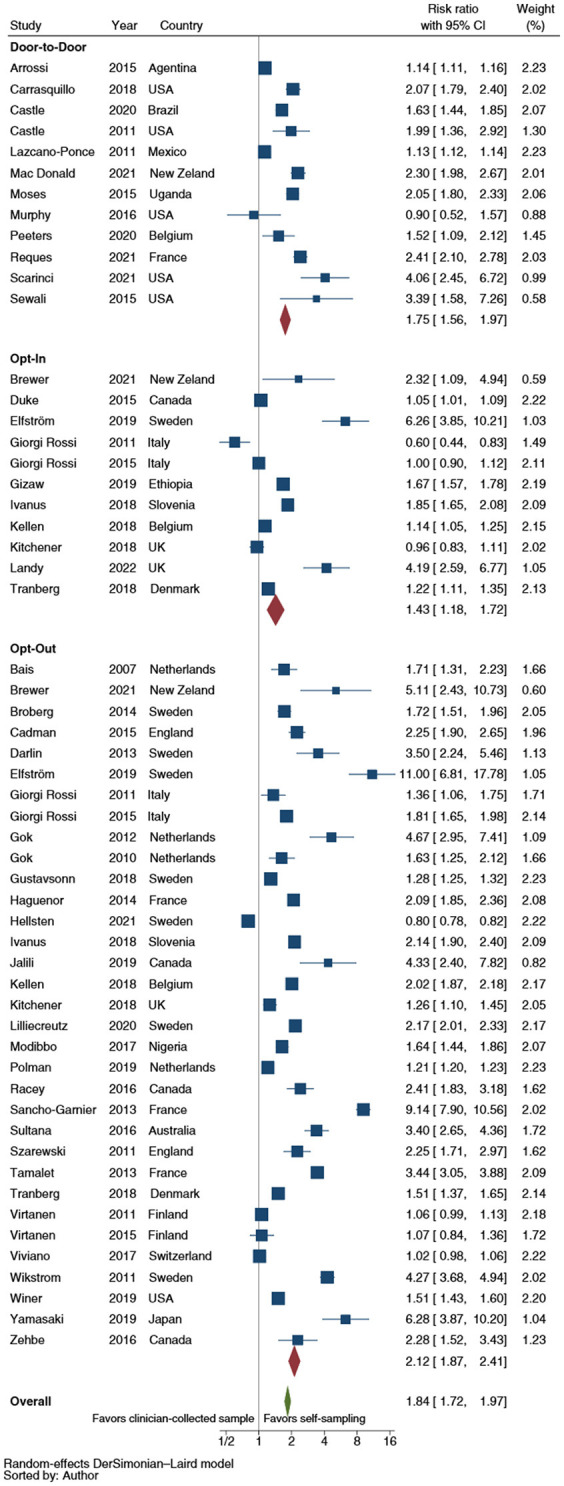
Forest plot comparing cervical cancer screening (CCS) uptake for HPV testing by strategy of self-samplers' distribution vs. clinician-collected samples. Homogeneity (*I*-squared): 98.8%; Cochrane's Q test for between-group differences: *Q* = 4,426.36; df = 2; *p* = 0.02.

With regard to self-sampler distribution strategy, the opt-out (RR: 2.1; 95% CI: 1.9–2.4) and the door-to-door (RR: 1.8; 95% CI: 1.6–2.0) did not statistically significant differ (*p* = 1.177) in improving the CCS uptake. In contrast, the opt-in (RR: 1.4; 95% CI: 1.2–1.7) showed a significantly lower efficacy than the opt-out strategy (*p* = 0.001); no statistically significant difference was displayed with respect to door-to-door distribution (*p* = 0.093) (Figure 3). The pooled analyses restricted to RCTs showed a statistically significant difference in improving CCS uptake between opt-out (RR: 2.2; 95% CI: 2.0–2.5) and door-to-door strategies (RR: 1.7; 95% CI: 1.5–2.0) (*p* = 0.048) and between the latter and the opt-in strategy (RR: 1.4; 95% CI: 1.1–1.7) (*p* = 0.048).

#### Device type

**Figure 4:**
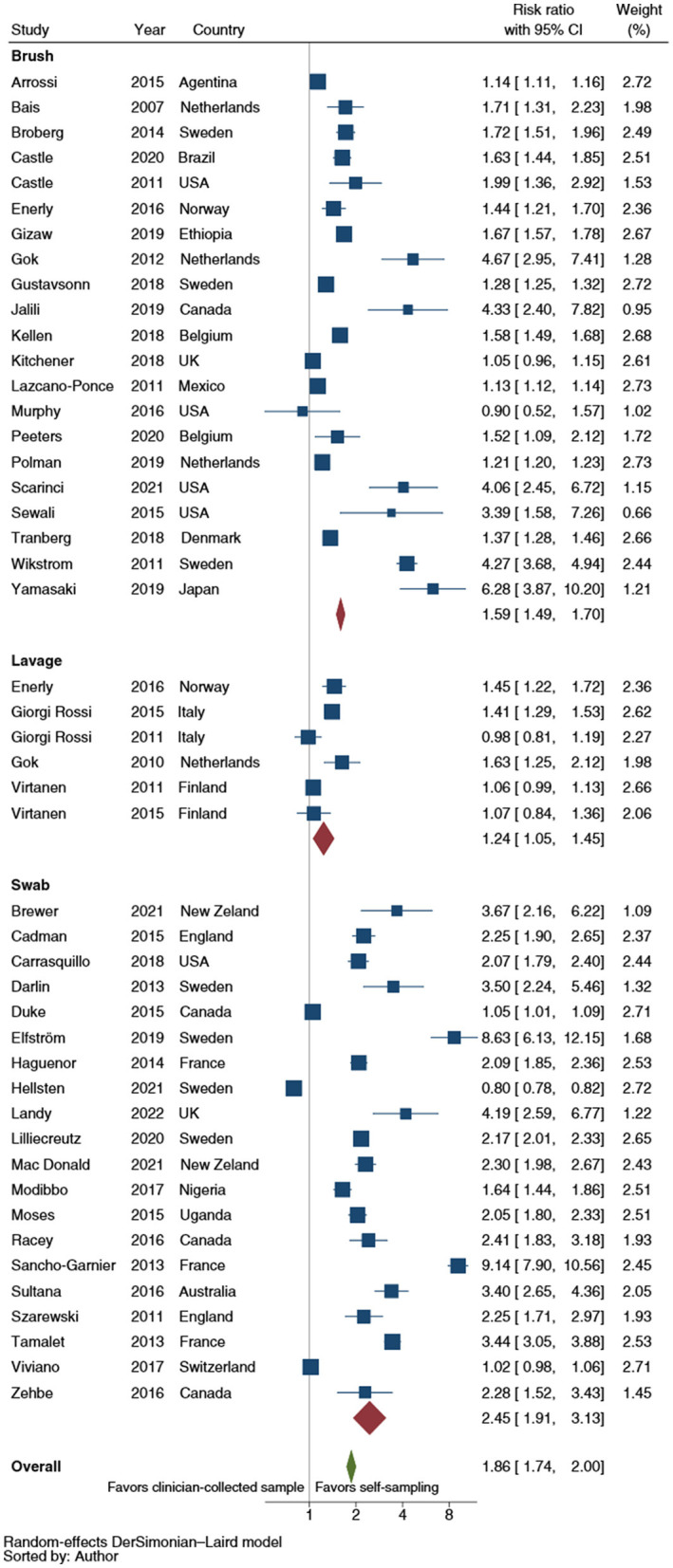
Forest plot comparing cervical cancer screening (CCS) uptake for HPV testing by self-sampler types vs. clinician-collected samples. Homogeneity (*I*-squared): 98.8%; Cochrane's Q test for between-group differences: *Q* = 3,904.90; df = 2; *p* = 0.02.

Figure 4 showed the RR of CCS uptake for HPV testing by self-sampler type. The results of those analyses showed a higher relative uptake for vaginal lavages (RR: 1.2; 95% CI: 1.1–1.5), brushes (RR: 1.6; 95% CI: 1.5–1.7) and swabs (RR: 2.5; 95% CI: 1.9–3.1) over clinician-collected samples. The analyses compared swabs and brushes and brushes and lavages showed a statistically significant difference (*p* = 0.004 and *p* < 0.001, respectively). When the analyses were restricted to RCTs, a pooled RR estimate of 2.7 (95% CI: 2.0–3.7) for swabs, 1.6 (95% CI: 1.5–1.7) for brushes and 1.3 (95% CI: 1.1–1.5) for lavages, were shown. Similarly, both the swabs-brushes (*p* < 0.001) and the brushes-lavages (*p* = 0.009) comparisons displayed a statistically significant difference.

#### Screening status

In the meta-analysis of studies reporting screening status, the overall RR was >1.00 indicating a potential effect of self-sampling in improving CCS uptake both among under-screened women (RR: 2.1; 95% CI: 1.9–2.3) and general population (RR: 1.4; 95% CI: 1.2–1.7) compared to clinician collected samples, and the difference was statistically significant (*p* < 0.001). Similarly, the efficacy of self-sampling was significantly higher (*p* = 0.015) when only RCTs were kept in the analysis, in both groups [under-screened women (RR: 2.1; 95% CI: 1.9–2.4) and general population (RR: 1.6; 95% CI: 1.3–1.9)].

#### Heterogeneity and publication bias

**Figure 5:**
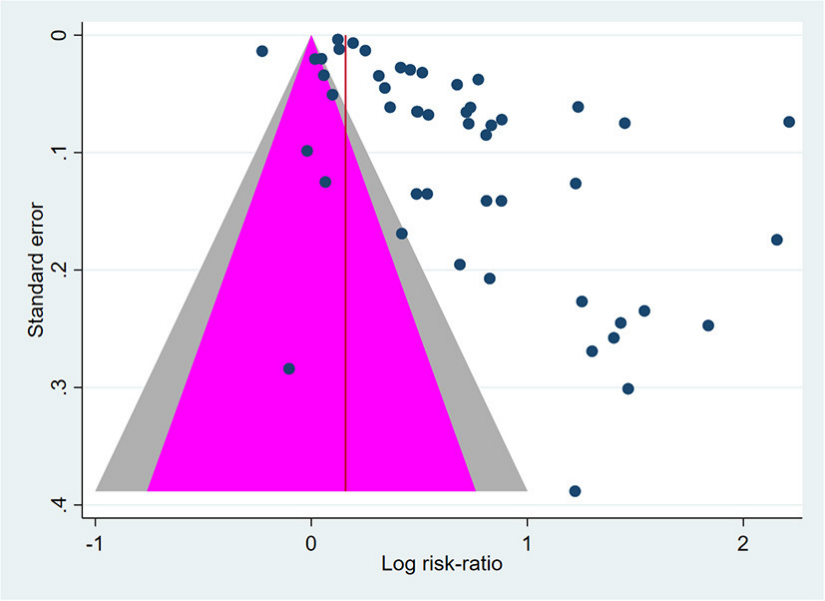
Contour-enhanced funnel plot of cervical cancer screening (CCS) uptake effect size (log odds-ratio) vs. Standard error. Outcome: screening uptake. Pink-area: *p* > 0.05. Gray area: 0.01 < *p* < 0.05. Blue dots represent single studies. Peters' test for publication bias: *p* = 0.060.

The level of heterogeneity was consistently high (*I*^2^ > 95%) in the overall and subgroup analyses. Publication bias was unlikely, as suggested by Peters' test (*p* = 0.06) (Figure 5).

### Secondary outcomes

Characteristics of the included studies assessing acceptability and preference of self-sampling vs. clinician-collected samples were displayed in Table 4. One-hundred and eight (70.1%) studies measured at least one secondary outcome: 12 (11.1%) of them were RCTs, 68 (63.0%) were cross-sectional studies and 28 (25.9%) had a quasi-experimental design. Seventy-two (66.7%) considered under-screened women, the rest involved the general population. Twenty-eight (25.9%) studies assessed acceptability and in 52 (48.2%) studies women were asked for preference. Both, acceptability and preference, were assessed in 28 (25.9%) studies. In 64 (59.3%) studies self-sampling occurred in a clinical setting, in 39 (36.1%) it occurred at home, and in 4 studies (3.7%) it occurred in both settings. The setting was not reported in one study.

**Table 4:** Characteristics of the included studies assessing acceptability and preference of self-sampling vs. clinician-collected samples.

**First authors**	**Year**	**Country**	**Design**	**Screening status**	**Age**	**Country economic status**	**Area**	**Social subgroup**	**Device type**	**Sampling setting**	**Total responders (acceptability)**	**Total responders (preference)**
Abdullah et al. (165)	2018	Malesia	Cross-sectional	General population	40.6*	MIC	Urban and rural	–	Brush	Clinic	164	164
Agorastos et al. (78)	2005	Greece	Quasi-experimental trial	Under-screened	44*	HIC	Urban and rural	–	Brush	Clinic	–	379
Aiko et al. (79)	2017	Japan	Quasi-experimental trial	Under-screened	40–49^†^	HIC	Urban	–	Brush	Home	–	127
Allende et al. (80)	2019	Bolivia	Cross-sectional	Under-screened	20–49^§^	MIC	Urban and rural	–	Brush	Clinic	–	221
Anderson et al. (81)	2017	USA	Cross-sectional	General population	44^#^	HIC	Urban and rural	Low-income women from North Carolina	Brush	Home	227	–
Anhang et al. (82)	2006	USA	Cross-sectional	Under-screened	35–44^†^	HIC	Urban	–	Swab	Clinic	–	172
Avian et al. (166)	2022	Italy	Quasi-experimental trial	General population	40–49^†^	HIC	Urban and rural	–	Swab	Clinic	–	1, 032
Bansil et al. (83)	2014	India, Nicaragua, Uganda	Cross-sectional	Under-screened	44*	MIC	Urban and rural	–	Brush	Clinic	–	3, 464
Barbee et al. (84)	2010	USA	Cross-sectional	Under-screened	18–70^§^	HIC	Urban and rural	Haitian immigrant women residing in Little Haiti	Swab	Home	245	245
Behnke et al. (85)	2020	Ghana	Cross-sectional	Under-screened	41*	MIC	Rural	–	Brush	Clinic	–	52
Berner et al. (86)	2013	Cameroon	Quasi-experimental trial	Under-screened	39^#^	MIC	Urban and rural	–	Swab	Clinic	–	217
Bosgraaf et al. (28)	2014	Netherlands	Randomized clinical trial	General population	44.5*	HIC	Urban	–	Brush and Lavage	Clinic	–	9, 360
Brewer et al. (87)	2019	New Zealand	Quasi-experimental trial	General population	30–69^§^	HIC	Urban and rural	–	Lavage and Swab	Clinic	–	44
Broquet et al. (88)	2015	Madagascar	Cross-sectional	General population	42, 5^##^	LIC	Urban and rural	–	Swab	Clinic	300	300
Castell et al. (89)	2014	Germany	Cross-sectional	Under-screened	53^#^	HIC	Urban and rural	–	Lavage	Home	108	–
Catarino et al. (34)	2015	Switzerland	Randomized clinical trial	General population	42^#^	HIC	Urban	–	Brush and Swab	Clinic	–	126
Catarino et al. (90)	2015	Switzerland	Cross-sectional	General population	43.6*	HIC	Rural	–	Swab	Home	130	–
Chatzistamatiou et al. (14)	2020	Greece	Cross-sectional	Under-screened	45^#^	HIC	Rural	–	Swab	Clinic	–	12, 376
Chatzistamatiou et al. (91)	2017	Greece	Cross-sectional	General population	44^#^	HIC	Rural	–	Brush	Clinic	339	334
Chaw et al. (167)	2022	Brunei	Cross-sectional	Under-screened	45^#^	HIC	Urban	–	Swab	Clinic	97	97
Chou et al. (92)	2016	Taiwan	Cross-sectional	General population	48^#^	HIC	Urban	–	Brush	Home	282	–
Crofts et al. (93)	2015	Cameroon	Cross-sectional	Under-screened	43^#^	MIC	Rural	–	Swab	Clinic	–	86
Crosby et al. (94)	2015	USA	Cross-sectional	Under-screened	40.2*	HIC	Rural	Rural appalachian women	Swab	Home	–	400
Dannecker et al. (95)	2004	Germany	Cross-sectional	Under-screened	42*	HIC	Urban	–	Brush	Clinic	333	318
de Melo Kuil et al. (96)	2017	Brasil	Quasi-experimental trial	Under-screened	25–45^†^	MIC	Urban and rural	–	Lavage	Clinic	–	160
Delerè et al. (97)	2011	Germany	Cross-sectional	Under-screened	25.7^##^	HIC	Urban	–	Lavage	Home	–	156
Des marais et al. (98)	2019	USA	Quasi-experimental trial	Under-screened	45^#^	HIC	Urban	–	Brush	Clinic and Home	188	–
Desai et al. (99)	2020	Nigeria	Cross-sectional	Under-screened	35–39^†^	MIC	Urban and rural	–	Brush	Clinic	–	9, 065
Duke et al. (100)	2015	Canada	Quasi-experimental trial	Under-screened	45–49^†^	HIC	Rural	–	Swab	Home	168	–
Dutton et al. (101)	2020	Australia	Cross-sectional	General population	35–39^†^	HIC	Rural	Aboriginal community	Swab	Home	200	–
Dzuba et al. (102)	2002	Mexico	Quasi-experimental trial	Under-screened	43*	MIC	Urban and rural	–	Swab	Clinic	–	1, 067
Esber et al. (168)	2018	Malawi	Cross-sectional	General population	33**	LIC	Rural	–	Swab	Clinic	199	199
Flores et al. (36)	2021	Mexico	Randomized clinical trial	General population	43.8*	MIC	Urban	–	Brush	Clinic	500	–
Galbraith et al. (104)	2014	USA	Cross-sectional	Under-screened	40–49^†^	HIC	Urban and rural	Women living in a situation of economic hardship	Brush	Home	211	211
Giorgi Rossi et al. (37)	2011	Italy	Randomized clinical trial	General population	25–64^§^	HIC	Urban and rural	–	Lavage	Home	–	139
Goldstein et al. (105)	2020	China	Quasi-experimental trial	General population	35–65^§^	HIC	Rural	–	Swab	Clinic	600	600
Gottschlich et al. (106)	2019	Thailand	Cross-sectional	Under-screened	50.44*	MIC	Urban and rural	–	Swab	Clinic	267	219
Gottschlich et al. (15)	2017	Guatemala	Cross-sectional	Under-screened	34.5*	MIC	Urban and rural	Indigenous community	Swab	Home	178	–
Guan et al. (107)	2012	China	Cross-sectional	Under-screened	41^#^	HIC	Rural	–	Brush	Clinic	–	174
Guerra Rodriguez et al. (169)	2022	Mexico	Cross-sectional	General population	26*	MIC	Urban	–	Brush	Clinic	60	60
Haile et al. (108)	2019	Ethiopia	Quasi-experimental trial	Under-screened	32*	LIC	Urban	–	Brush	Clinic	83	83
Harper et al. (44)	2002	USA	Randomized clinical trial	Under-screened	37.7*	HIC	Urban	–	Swab and Tampon		67	–
Hinten et al. (109)	2017	Holland	Cross-sectional	Under-screened	56^#^	HIC	Urban	Renal transplant recipients women	Brush	Clinic	–	157
Igidbashian et al. (110)	2011	Italy	Quasi-experimental trial	Under-screened	38^#^	HIC	Urban	–	Brush and Lavage	Clinic	–	Lavage: 76 Brush: 96
Ilangovan et al. (111)	2016	USA	Cross-sectional	Under-screened	52*	HIC	Urban	Latina and Haitian patients	Swab	Clinic	120	120
Islam et al. (112)	2020	Kenia	Quasi-experimental trial	Under-screened	39^#^	MIC	Urban	Sex Workers	Brush	Clinic	–	399
Jones et al. (113)	2012	United States	Quasi-experimental trial	General population	45^#^	HIC	Urban	–	Lavage	Clinic	–	197
Jones et al. (114)	2008	Netherlands	Cross-sectional	Under-screened	35^#^	HIC	Urban	–	Lavage	Home	–	91
Karjalainen et al. (48)	2016	Finland	Randomized clinical trial	Under-screened	40–49^†^	HIC	Urban and rural	–	Brush and Lavage	Clinic	–	Lavage: 161 Brush: 159
Katanga et al. (115)	2021	Tanzania	Quasi-experimental trial	Under-screened	41*	LIC	Urban	–	Brush	Home	–	416
Ketelaars et al. (116)	2017	Netherlands	Quasi-experimental trial	Under-screened	43.4*	HIC	Urban	–	Brush	Clinic	–	2, 131
Khanna et al. (117)	2007	USA	Quasi-experimental trial	Under-screened	32*	HIC	Urban	–	Brush	Clinic	–	499
Khoo et al. (12)	2021	Malaysia	Cross-sectional	Under-screened	35–45^§^	MIC	Urban	–	Swab	Clinic	725	725
Kilfoyle et al. (118)	2018	USA	Cross-sectional	General population	44^#^	HIC	Urban and rural	Low-income women from North Carolina	Brush	Home	–	221
Kohler et al. (13)	2019	Botswana	Cross-sectional	Under-screened	45*	MIC	Urban and rural	–	Swab	Clinic	104	105
Landy et al. (119)	2022	UK	Cross-sectional	General population	55–59^†^	HIC	Urban	–	Brush	Clinic	–	170
Laskow et al. (120)	2017	El Salvador	Cross-sectional	General population	40.7*	MIC	Rural	–	Brush	Home	41	–
Litton et al. (121)	2013	USA	Cross-sectional	Under-screened	35.4**	HIC	Rural	African American women living in the Mississippi Delta	Swab	Home	–	516
Lorenzi et al. (122)	2019	Brasile	Cross-sectional	Under-screened	36.2*	MIC	Urban	–	Brush	Clinic	–	116
Madhivanan et al. (124)	2021	India	Cross-sectional	Under-screened	39^#^	MIC	Rural	–	Brush	Clinic	118	118
Mahande et al. (125)	2021	Tanzania	Cross-sectional	General population	35.6*	LIC	Urban and rural	–	Swab	Home	350	–
Malone et al. (126)	2020	USA	Cross-sectional	General population	40–49^†^	HIC	Urban	–	Swab	Home	–	117
Mandigo et al. (127)	2015	Haiti	Cross-sectional	General population	18–50^§^	LIC	Rural	–	Not Reported	Home	485	–
Mao et al. (128)	2017	USA	Cross-sectional	Under-screened	35.7*	HIC	Urban	–	Swab	Home	–	1, 759
Ma'som et al. (123)	2016	Malaysia	Cross-sectional	Under-screened	38^#^	MIC	Urban	–	Brush	Clinic	–	803
Maza et al. (129)	2018	El Salvador	Cross-sectional	General population	42.86*	MIC	Rural	–	Not Reported	Home	1, 867	–
McLarty et al. (130)	2019	USA	Cross-sectional	Under-screened	49^#^	HIC	Urban	–	Tampon	Home	–	55
Molokwu et al. (55)	2018	USA	Randomized clinical trial	Under-screened	46.4*	HIC	Urban and rural	Border dwelling hispanic women	Swab	Home	–	107
Mremi et al. (131)	2021	Tanzania	Cross-sectional	General population	35–44^†^	LIC	Urban and rural	–	Swab	Home	1, 108	–
Murchland et al. (11)	2019	Guatemala	Cross-sectional	Under-screened	33.9**	MIC	Rural	–	Swab	Home	760	–
Nakalembe et al. (132)	2020	Uganda	Cross-sectional	Under-screened	34#	LIC	Rural	–	Brush	Clinic	1, 316	–
Nelson et al. (133)	2015	USA	Quasi-experimental trial	Under-screened	24.1**	HIC	Rural	–	Swab	Home	–	62
Ngu et al. (170)	2022	Hong Kong	Quasi-experimental trial	Under-screened	43^#^	HIC	Urban	–	Swab	Home	295	–
Nobbenhuis et al. (134)	2002	Holland	Quasi-experimental trial	General population	35*	HIC	Urban	–	Lavage	Clinic	–	56
Obiri-Yeboah et al. (135)	2017	Ghana	Quasi-experimental trial	Under-screened	44.1*	MIC	Urban	–	Brush	Home	–	194
Oranratanaphan et al. (136)	2014	Thailand	Quasi-experimental trial	Under-screened	40.6*	MIC	Urban	–	Brush	Clinic	–	100
Pantano et al. (137)	2021	Brazil	Cross-sectional	Under-screened	49.4*	MIC	Urban and rural	–	Brush	Home	405	313
Penaranda et al. (138)	2015	USA	Cross-sectional	Under-screened	48.2*	MIC	Urban and rural	Border dwelling women	Swab	Clinic	118	106
Polman et al. (59)	2019	Holland	Randomized clinical trial	Under-screened	43.7*	HIC	Urban and rural	–	Brush	Clinic	–	1, 662
Racey et al. (16)	2016	Canada	Randomized clinical trial	General population	51.2**	HIC	Rural	–	Swab	Home	68	–
Reiter et al. (139)	2020	USA	Cross-sectional	General population	46, 7*	HIC	Urban	–	Tampon	Home	79	79
Rosenbaum et al. (140)	2014	El Salvador	Cross-sectional	Under-screened	41–59^†^	MIC	Rural	–	Brush	Clinic	–	518
Sellors et al. (142)	2000	USA	Quasi-experimental trial	Under-screened	31.5*	HIC	Urban	–	Brush	Home	127	–
Shin et al. (143)	2019	Korea	Cross-sectional	Under-screened	20–49^†^	HIC	Urban	–	Swab	Clinic	728	–
Sechi et al. (141)	2022	Italy	Quasi-experimental trial	Under-screened	39, 5*	HIC	Urban	–	Swab	Clinic	40	–
Silva et al. (144)	2017	Portugal	Cross-sectional	Under-screened	26*	HIC	Urban	–	Not Reported	Not Reported	303	276
Sormani et al. (171)	2022	Cameroon	Cross-sectional	General population	40.6^#^	MIC	Urban	–	Swab	Clinic	2, 196	2, 201
Surriabre et al. (145)	2017	Bolivia	Cross-sectional	Under-screened	25–59^§^	MIC	Urban and rural	–	Not Reported	Clinic	–	201
Swanson et al. (146)	2018	Kenya	Cross-sectional	General population	36*	MIC	Rural	–	Tampon	Home	255	–
Szarewski et al. (147)	2007	UK	Quasi-experimental trial	Under-screened	32^##^	HIC	Urban	–	Swab	Clinic	–	702
Taku et al. (148)	2020	South Africa	Cross-sectional	Under-screened	44^##^	MIC	Rural	–	Brush	Clinic	737	720
Tan et al. (149)	2021	Malesia	Quasi-experimental trial	General population	40.5*	MIC	Urban and rural	–	Brush	Clinic	10	10
Tiiti et al. (150)	2021	Sud Africa	Cross-sectional	General population	36.8*	MIC	Urban and rural	–	Brush and Swab	Clinic	526	526
Torrado Garcia et al. (151)	2020	Colombia	Cross-sectional	Under-screened	46.5^#^	MIC	Urban	Women belonging to the low socioeconomic stratum	Brush	Clinic	420	420
Torres et al. (152)	2018	Brasile	Cross-sectional	Under-screened	26–36^†^	MIC	Rural	–	Brush	Home	–	412
Trope et al. (153)	2013	Thailand	Cross-sectional	Under-screened	25–60^§^	MIC	Rural	–	Swab	Clinic	388	388
Van Baars et al. (154)	2012	Netherlands	Cross-sectional	Under-screened	40*	HIC	Urban	–	Brush	Clinic	127	–
Van de Wijgert et al. (68)	2006	South Africa	Randomized clinical trial	Under-screened	29.9*	MIC	Urban	–	Swab and Tampons	Clinic	–	Swab: 222 Tampon: 228
Virtanen et al. (155)	2014	Finland	Cross-sectional	General population	40–49^†^	HIC	Urban and rural	–	Lavage	Home	809	889
Waller et al. (17)	2006	UK	Quasi-experimental trial	Under-screened	34.2*	HIC	Urban	–	Swab	Clinic	–	902
Wang et al. (156)	2020	USA	Cross-sectional	Under-screened	50^#^	HIC	Urban	HIV positive women	Brush	Clinic and Home	61	–
Wedisinghe et al. (157)	2022	Scotland	Quasi-experimental trial	General population	51.9**	HIC	Rural	–	Brush	Clinic and Home	272	–
Wikstrom et al. (158)	2007	Sweden	Cross-sectional	General population	35–44^†^	HIC	Urban and rural	–	Swab	Home	–	91
Winer et al. (159)	2016	USA	Cross-sectional	Under-screened	43*	HIC	Rural	–	Swab	Clinic and Home	318	306
Wong et al. (74)	2018	Hong Kong	Randomized clinical trial	Under-screened	38.2*	HIC	Urban	Sex workers	Swab	Clinic	–	68
Wong et al. (160)	2020	Hong Kong	Cross-sectional	General population	39*	HIC	Urban	–	Brush	Home	–	124
Wong et al. (75)	2016	Hong Kong	Randomized clinical trial	Under-screened	50.9*	HIC	Urban	–	Swab	Clinic	351	392
Zehbe et al. (161)	2011	Canada	Cross-sectional	Under-screened	25–39^†^	HIC	Rural	Women belonging to the First Nation community	Swab	Clinic	47	48

Sample age reported as

* mean,

** weighted mean,

# median,

## weighted median,

† median age group or

§ range.

Country economic status reported as: HIC, high income country; MIC, middle income country; LIC, low income country.

#### Acceptability

**Figure 6:**
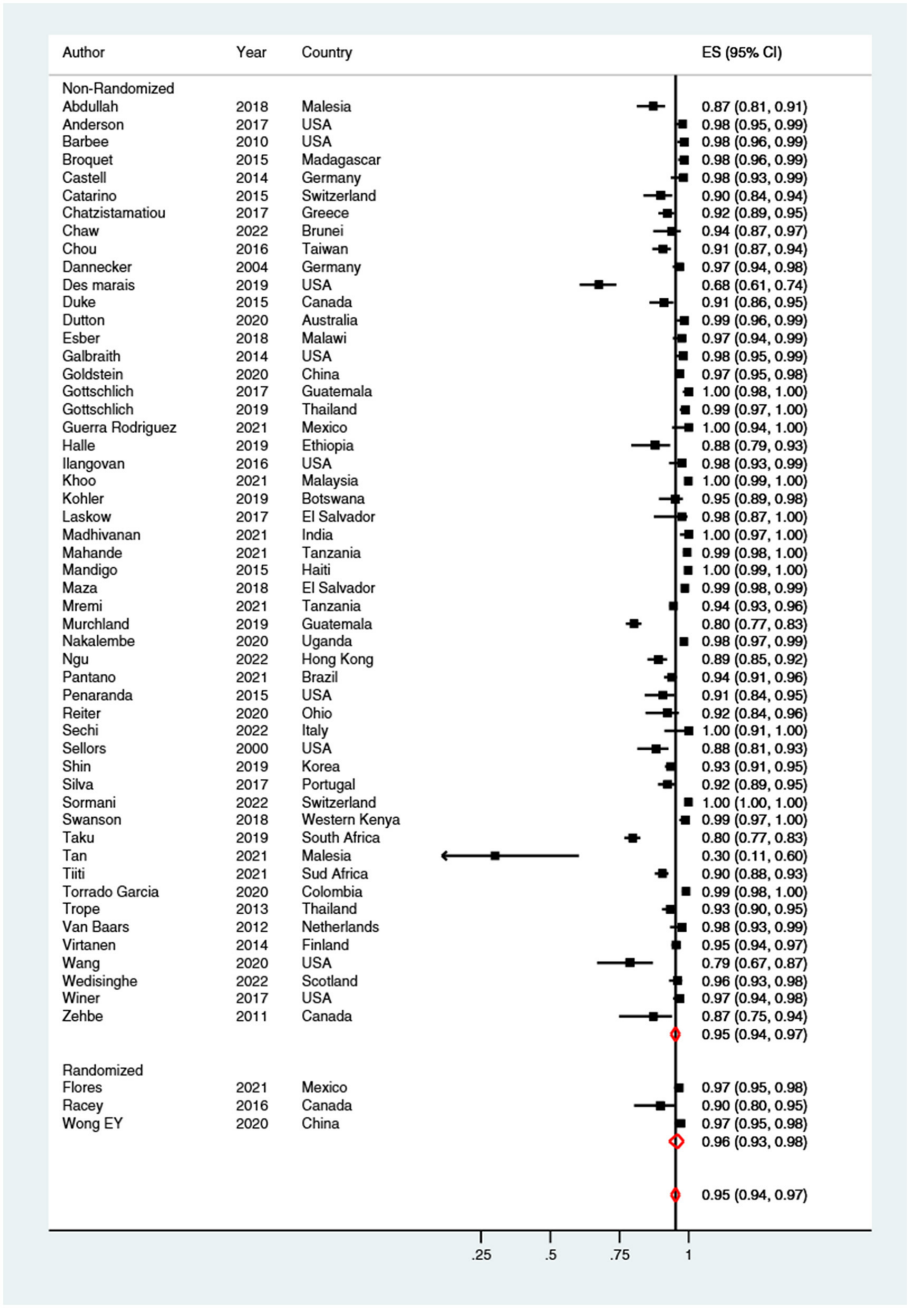
Forest plot of the proportion of women who found self-sampling acceptable. Homogeneity (*I*-squared): 95.9%; Cochrane's Q test for between-group differences: *Q* = 1,307.30; df = 54; *p* < 0.001.

Meta-analyses examining the proportion of women who found self-sampling acceptable, showed a very high pooled estimate (95%; 95% CI: 94–97%) (Figure 6). No differences (*p* = 0.420) were found among acceptability of brushes (93%; 95% CI: 90–96%), swabs (96%; 95% CI: 93–98%), lavages (98%; 95% CI: 95–100%) and tampons (97%; 95% CI: 92–100%). Moreover, the percentage of women who self-reported acceptance of self-sampling at home (96%; 95% CI: 93–98%) overlapped with acceptance of self-sampling in a clinical setting (96%; 95% CI: 94–98%). In all meta-analyses high heterogeneity (*I*^2>^ 95%) was observed.

#### Preference

**Figure 7:**
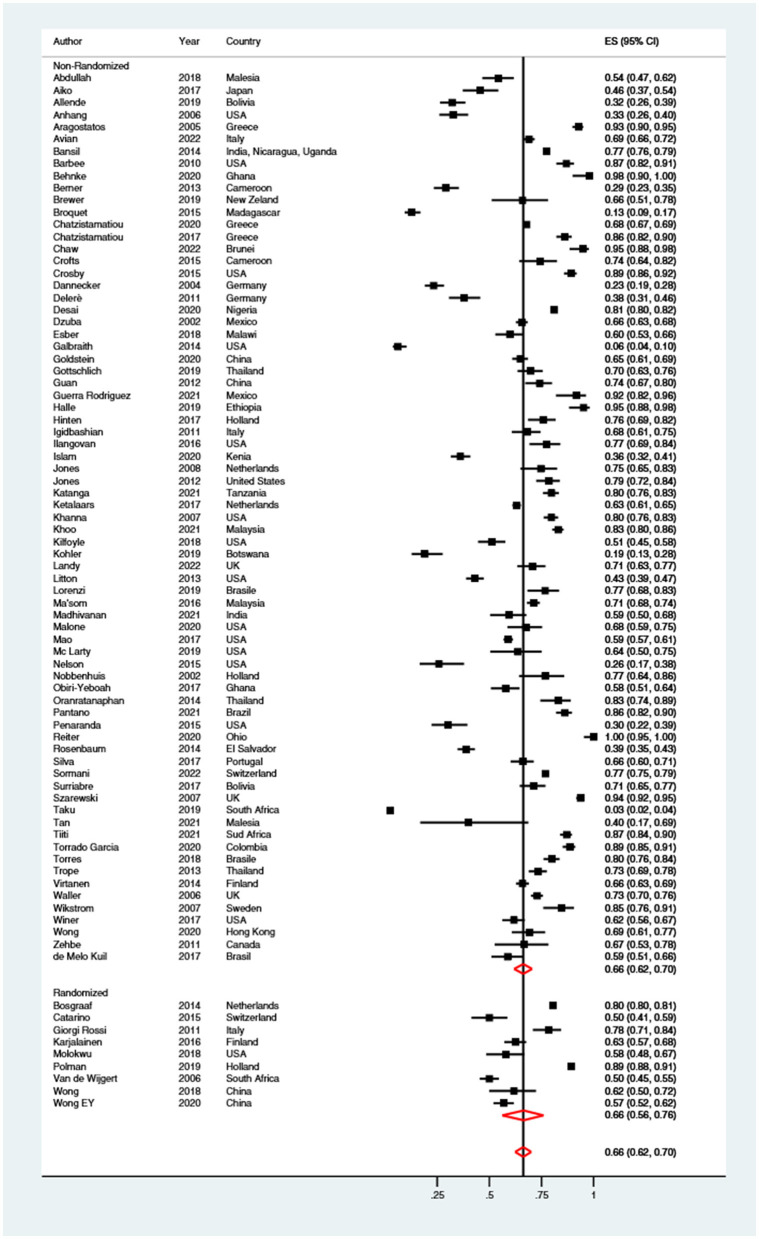
Forest plot of the proportion of women preferring self-sampling over clinician-collected samples. Homogeneity (*I*-squared): 99.0%; Cochrane's Q test for between-group differences: *Q* = 7,842.51; df = 81; *p* < 0.001.

Sixty-six percent (95% CI: 62–70%) of women preferred self-sampling procedures vs. clinician-collected samples (Figure 7). No significant difference (*p* = 0.850) was shown when brushes (67%; 95% CI: 58–74%), swabs (65%; 95% CI: 59–70%), lavages (68%; 95% CI: 60–76%) and tampons (77%; 95% CI: 31–100%) were compared. Finally, the preference of women for self-sampling was almost equal (*p* = 0.841) when it was performed at home (66%; 95% CI: 57–74%), or in a clinical setting (67%; 95% CI: 62–71%). The level of heterogeneity was high (*I*^2>^ 95%).

## Discussion

The findings of the present meta-analysis provide a summary of the implementation options of self-sampling for HPV testing. Since the COVID-19 pandemic has had an enormous impact on CCS attendance, self-sampling could offer a unique opportunity for catch-up screening and will play an important role in improving the global coverage of CCS. Indeed, the World Health Organization strongly recommends the use of self-sampling for HPV screening to contribute to reaching a coverage of 70% by 2030 and eliminate HPV correlated diseases in the next decades (172). Considering that for an intervention to be effective it must be broadly accepted, evidence about women's acceptability for CCS comparing self-sampled with clinician-collected specimens is also provided.

The findings of the present meta-analysis showed that self-sampling for HPV testing is an effective tool to reach women in the context of organized CCS programs. Indeed, women were nearly twice as likely to use CCS services through self-sampling as compared with clinician-based sampling. Considering that the option of cervical precancer detection from self-collected samples showed similar clinical accuracy for hrHPV testing as clinician-collected samples (9, 173, 174), this result increases evidence in support of incorporating self-sampling into organized screening programs to better respond to the disruption of CCS programs after the COVID-19 pandemic. Moreover, the meta-analyses split into sub-groups according to dissemination strategies, suggested that a door-to-door approach, in which an HCP visits women at home to inform on CCS and offer a self-sampling HPV test kit, has almost doubled the CCS uptake by seven-fold. However, it has to be pointed out that the door-to-door approach has been mainly investigated in low-resource settings or for reaching under-screened women in high-resource settings. The findings showed an even higher likelihood of attending CCS for the opt-out approach (i.e., mailing of self-collection devices to women's homes without them taking the initiative), compared with controls (i.e., invitation letters sent home, reminding phone calls or suggestions from the HCP to be screened in the local hospital or from a gynecologist). In high-resource settings, research has focused on an alternative invitation scenario (opt-in strategy) in which women request a self-collection kit that is mailed to home or pick it up at pharmacy or clinic. The analyses showed that the opt-in approach reached a high CCS uptake when compared to mailing a reminder letter proposing a clinician-collected samples, although lower than response rates to the opt-out and door-to-door approaches. It should be noted that the opt-in approach has the advantage to be less expensive, especially on a national level. Bring together, these results confirm recent literature. In particular, the meta-analysis by Yeh et al., found that opt-out strategy increased CCS participation (RR: 2.27; 95% CI: 1.89–2.71) (19), and Arbyn et al. found similar results when comparing opt-out self-samplers distribution with a reminder letter/advice from HCP to have a clinician to collect the sample (9).

In the relevant studies, several types of devices to collect exfoliated cells of the cervicovaginal duct for HPV-DNA detection were employed. It should be noted that the distribution of brush- and swab-based devices were associated with significantly higher uptake when compared with invitation to be sampled by a clinician. The latter result deserves attention since, as previously demonstrated, the type of HPV self-sampling device may play an important role in women's acceptability and preference of a CCS strategy (87, 110). The findings of the present meta-analysis highlighted high pooled acceptability and overall preference of self-sampling compared to clinician-based sampling, downsizing potential concerns about self-sampling (e.g., worry of not being able to correctly carry out the sampling), as previously described (17, 175, 176). The finding that especially non-attender women preferred self-sampling to clinician-based sampling for future CCS programs deserves attention, for its potential to increase participation in primary CCS. High acceptability and preference of self-sampling have the potential to improve CCS uptake and its effects on incidence and mortality from cervical cancer. Acceptability of self-sampling demonstrated advantages from both public health and individual patient perspective (177). Proper communication of the self-sampling process to women needs to be realized to address eventual women's concerns and emphasizes that most women are able to successfully obtain an adequate sample or deliver self-sampling by HCPs who can explain the process face-to-face.

In contrast to the findings of Nishimura et al., who documented that swabs were preferred by women when compared with other devices (10) no differences in acceptability regarding the type of self-sampling devices were found.

Contextual factors are essential in real life decision-making: when referring to a small community, offering a door-to-door device could be the most preferable strategy. Differently, when a high number of women have to be reached, mailing the device could represent a cost-effective alternative. Regarding the type of self-sampler device, a pilot investigation could be useful before introducing a large-scale use of self-samplers, as suggested by Arbyn et al. (9). Moreover, elements to consider in order to improve CCS uptake are cultural, religious and socio-economic characteristics of the target community (55, 178, 179). A study carried out on Nigerian women showing that individuals with greater spirituality were less likely to carry out self-sampling (180). Similarly, a systematic review focusing on Islamic women shows that cervical cancer prevention still represents a considerable taboo among them and this can lead to under-screening (181). Further, additional aspects that can interfere with the effectiveness of a self-sampling campaign are the perceived costs and time required for being screened (178, 179, 182). The costs and the need to inform women about the importance of being screened are pivotal among migrants and minorities (183). In the authors' opinion, the use of prepaid and pre-addressed envelopes, the absence of costs for women, the presence of clear and detailed instructions in the self-sampling kits and continuous education about the importance of CCS, could be decisive factors to maximize the uptake.

## Strengths and limitations

To the best of our knowledge no recent meta-analysis measuring the effect of self-sampling, across different distribution strategies, type of devices and screening status has been conducted, and the present results could be pivotal to provide practical suggestions for the organization of CCS program. Further strengths consist of the considerable number of subjects included, and the analysis of the recently published results of RCTs.

As above-mentioned, a possible limitation of this meta-analysis is the high heterogeneity, likely attributable to the wide socio-cultural diversity of the samples of women enrolled. Consequently, the results must be interpreted with caution highlighting the need to consider potential factors underlying the success of a self-sampling CCS campaign. Other limitations are the lack of search in the gray literature and the exclusion of all findings reported in languages different than English.

## Conclusions

Self-sampling has the potential to increase participation of under-screened women in the CCS, in addition to the standard invitation to have a clinician to collect the sample. For small communities door-to-door distribution could be preferred to distribute the self-sampler; while for large communities opt-out strategies should be preferred over opt-in. Finally, since no significant difference in acceptability and preference of device type was demonstrated among women, and swabs exhibited a potential stronger effect in improving CCS, these devices could be adopted primarily over tampons and lavages.

## Data availability statement

The original contributions presented in the study are included in the article material, further inquiries can be directed to the corresponding author.

## Author contributions

FL participated in the conception and design of the study, contributed to the data collection, and wrote the first draft of the article. GD participated in the conception and design of the study, collected the data, performed the data analysis, contributed to analysis interpretation, and wrote the first draft of the article. AT contributed to the data collection and to the data analysis. AB designed the study, was responsible for the data collection and interpretation, wrote the article, and was guarantor for the study. All authors take responsibility for the integrity of the data and the accuracy of the data analysis. All authors have read and approved the manuscript for publication.
